# Genomic testing in healthcare: a hybrid space where clinical practice and research need to co-exist

**DOI:** 10.1080/14737159.2019.1672540

**Published:** 2019-10-11

**Authors:** Rachel Horton, Anneke Lucassen

**Affiliations:** 1 Clinical Ethics and Law at Southampton (CELS), Faculty of Medicine, University of Southampton, UK; 2 Wessex Clinical Genetics Service, Princess Anne Hospital, Southampton, UK

**Keywords:** Ethics, research, clinical, genetic testing, genomics, consent

## Abstract

**Introduction**: Clinical practice and research are traditionally seen as distinct activities that are governed by different principles and processes. Innovative technologies such as genomic testing challenge this model, involving many activities that cannot be easily categorized as purely research, or purely clinical care.

**Areas covered**: We discuss the interdependence of research and clinical practice in the context of genomics, for example, when determining the significance of rare genetic variants, or diagnosing newly described rare diseases. We highlight the potential of the symbiotic relationship between clinical practice and research.

**Expert opinion**: In the context of genomics, it is not appropriate to treat clinical practice and research as entirely separable. Forcing binary categorization of activities as one or the other risks losing the many benefits that derive from their integration. We need to explore the hybrid area where clinical practice and research coincide, developing governance that allows us to maximize its potential, rather than insisting that hybrid clinical-research activities conform to processes built for ‘pure clinical practice’ or ‘pure research’. We argue the need for a renegotiation of the contract around genomic testing, recognizing, valuing and facilitating the hybrid space where clinical practice and research co-exist.

## 1. Introduction

Clinical practice and research are often regarded as separate enterprises, governed by different legislations, and representing different relationships between the parties involved [1]. In the context of clinical care, patients engage with clinicians to access information or treatment to help them cure, prevent, or live more easily with health conditions. Patients might provide potentially sensitive details, and allow possibly intrusive examination or investigations, on the premise that having such information is necessary in order for clinicians to recommend and provide appropriate care. The patient and clinician might negotiate a number of potential responses to an issue, talking through the risks and benefits of each course of action, aiming to reach agreement on how to proceed. Such interactions assume that clinicians will seek and protect patient information thoughtfully; and that patients will consider the opinions provided by their clinicians.

Patients usually engage with clinical care anticipating direct personal benefit (though other parties may drive this engagement, for example, a person’s family or friends might encourage them to see their GP about a concern). Clinical interactions tend to be framed around a clinician’s duty of care to a patient, with a presumption that only beneficial options would be offered. They typically only involve written contracts between clinician and patient in certain situations, such as to document consent to an operation.

The relationship between the research participant and researcher is somewhat different. Whilst ‘first do no harm’ is a principle that pervades clinical medicine, this maxim is given even greater primacy in the research environment. The bar for justification of participation in research is generally higher than for clinical care, as any potential benefits of engagement with research are uncertain or indirect, and many people engaging with research do so for altruistic motives, rather than because they themselves will benefit. Separate partitioning of research and clinical care (and the idea that they can easily uncouple) is often implied by statements such as ‘your clinical care will not be affected’ in participant information sheets and consent forms for research.

Research is often conceptualized as an optional extra in a way that engagement with clinical care is usually not. Clearly, ‘research’ is not a homogeneous activity: for example, some research is likely to have a profound direct physical impact on participants (such as taking part in a trial to evaluate a new kind of pacemaker) whereas the consequences of participation in other research will likely be more passive (for example, data collection using questionnaires). However, whatever the nature of the research, research governance tends to strongly emphasize the importance of ‘informed consent’, and often requires explicit demonstration that a participant has had time to consider whether to take part in an activity that might not benefit them. Whilst the extent of scrutiny is appropriately proportional to the invasiveness of the research, all research needs to satisfy formalized requirements around information provision and consent processes (or justify why this is not necessary). Such requirements arose partly in response to scandals such as the Tuskegee Syphilis Study, where participants were lied to and denied effective treatment in order to further the ends of the research [2].

There is nothing novel in the idea that research and clinical practice may be closely tied. For example, people participating in a research trial testing out a medication might benefit, or experience side-effects, from the drugs that they are given in the course of the trial. Similarly, the clinical care provided to patients now draws on evidence from research done in the past. Historically in such scenarios, defining a boundary between research and clinical care has been (at least superficially) simple: a person can stop taking the research drug; a drug developed through research can be licensed for prescription in the clinic. Even here, the boundaries between research and clinical care are increasingly indistinct. For example, the drug nusinersen was made available to patients with spinal muscular atrophy who trialed it in the context of research projects, beyond the point at which the research finished [3]. In the UK, the initial NICE consultation did not recommend nusinersen for NHS funding, because ‘*there is no long-term evidence, so the long-term benefits are highly uncertain*’ and ‘*there are also important limitations and uncertainties in the economic evidence*’ [4]. More recently a Managed Access Agreement was developed allowing many people with spinal muscular atrophy to access nusinersen whilst data collection is ongoing regarding its effectiveness [5]. In diagnostic genomic testing, boundaries between research and clinical care may be blurred as a matter of course – its complex and exploratory nature means that these enterprises may be necessarily entangled.

## 2. The interdependence of clinical practice and research in genomic testing

Genomic testing works by trawling a person’s genetic code, picking out the four to five million genomic variants that each person has, then filtering them in order to produce a meaningful output. These challenges and exposes our ignorance about what much of our genetic code actually means – more links between genes and disease are identified every year [6], and previous ideas about how and whether particular variants are linked to disease are sometimes overturned [7]. Whilst a person’s genetic code is largely fixed throughout their life, interpretations of it may be in flux [8]. Differing ‘results’ from the same person’s genome might arise due to different clinical questions being asked of the same raw data, but might also reflect a learning process where updated evidence is brought to bear in the interpretation process. Further questions arise as to how far consent can guide this process: in the context of genomic testing, patients and clinicians might agree as to what sorts of information they intend to look for within the genetic code but it is not possible to specify upfront exactly what might be found [9].

The technical ability to identify genetic variants, and understanding what these variants might mean for a patient, are not the same but are sometimes dangerously conflated. Both elements are required for a useful test. Variant identification is now comparatively simple: genomic testing can detect a patient’s genome sequence with a high degree of accuracy and reliability. However, variant interpretation is not a simple bolt-on to the technical sequencing process, but a complex undertaking which combines knowledge of the clinical context, understanding of relevant research, and collaborative learning from previous genomic tests, in order to reach a view as to the medical importance (or not) of a particular variant [10]. Whilst variant identification by genome sequencing is now operating at a level suitable for adoption in the clinic, variant interpretation cannot responsibly be uncoupled from research.

In interpreting the significance of a variant identified via genomic testing, scientists and clinicians will draw on a range of research evidence, such as the variant’s presence in databases of normal variation, or its having been previously reported in association with the disease [11]. For well-understood variants, this process can be seen as compatible with a conventional clinical care model, comparable to a clinician looking up research evidence in order to decide whether to suggest a particular medication for a patient’s symptoms (though recent evidence from population studies should shake our confidence in our ability to interpret even ‘well-understood’ genetic variants in unfamiliar contexts [12]). However, the clinical significance of genetic variants is often uncertain. Here, clinicians might try to resolve uncertainty by testing other people in a family for the same variant to see if the inheritance pattern looks plausible, or by arranging functional studies to see if the variant seems to be affecting the way that the gene works in practice [10]. Such activities might allow increasing confidence as to whether the variant is responsible for a patient’s health problems, and with clarity might come clinical options like tailored treatments, or prenatal testing.

A further example of the interdependence between clinical care and research arises from studies like the Deciphering Developmental Disorders study and the 100,000 Genomes Project [13,14]. These regularly identify new genes implicated in rare genetic conditions by analyzing and comparing the genomes of different people with similar rare symptoms and features [15]. Patients with variants in such genes might be invited to appointments with their clinicians to discuss them, but these appointments often involve a mixture of explaining in tentative terms what a new diagnosis might mean for a patient, whilst at the same time learning from the patient what clinical features variants in this gene might give rise to [16]. A possible ‘clinical diagnosis’ has been identified through research, but the learning process as to what this diagnosis means has only just started.

We argue that patients and clinicians are not shuttling between pure clinical and pure research activities when exploring what genetic variants mean – understanding of genetic variants becomes possible because elements of both clinical care and research co-exist. In asking a research team to perform a functional test that could ratify a clinical diagnosis, or in carefully phenotyping a person with variants disrupting a putative new disease gene, patients and clinicians are undertaking both. Governance conventions might currently require that clinical care and research are treated as if they are dichotomous, but in practice, some activities cannot be neatly pigeonholed as one or the other.

Learning health-care systems are a potentially useful model here. These involve continuous feedback loops where new knowledge is derived from clinical care, and used to inform and improve future care [17]. They rely on the premise that the process of delivering care provides in itself a useful opportunity to learn how to better provide care in the future [18]. Implementation of learning health-care systems have been closely tied to the uptake of electronic medical records due to their potential to collect large volumes of data in a format amenable for analysis. Genome sequences are an example of data-rich information that could be held within electronic medical records. The value of genomic datasets as a substrate for ongoing learning will rely on individual genomic sequences being tied to accurate and regularly updated phenotypic information, as well as wider genomic context, and clearly this has potential ramifications for participant privacy that need to be considered.

However, the hybrid nature of genomic testing again presents a potential challenge: when is knowledge learnt by a learning healthcare system ‘ready’ to be used in the clinic? The thresholds might be different in different situations – for example, in the context of rare disease diagnosis, it might be appropriate to communicate information of a relatively tentative nature in the process of establishing or refuting a diagnosis. However, at a broader public health level, we might require that insights from a learning healthcare system are much more certain before being used to inform clinical care. This raises questions as to how long a learning healthcare system can learn for before having an obligation to directly link that learning with clinical practice. For example, population analyses are demonstrating that many genomic variants previously considered ‘pathogenic’ or causative, are much less penetrant when found outside the context of a family history of the relevant disease (and thus explain only part of the ‘causation’ of the phenotype) [12]. Genomics-tailored learning health-care systems might provide a useful opportunity to learn the penetrance of such variants in a population setting, but is it acceptable for them to learn this from people who have those genetic variants, without informing them that they have a variant regarding which the clinical consequences are uncertain, and interpretations of ‘effect’ may still go up or down?

Whilst genomic testing evidently has both clinical and research dimensions, without appropriate regulation, the liminal space where research and clinical care should co-exist [19] is treated as a no man’s land. Hybrid activities are forced either to pick a side, or to contort themselves in order to conform to the potentially contradictory requirements of both ‘pure research’ and ‘pure clinical care’. For example, the consent form suggested by Genomics England for genomic testing has gone to a Research Ethics Committee for approval. However, the intention is that these forms will be completed within standard clinic appointments, so that the time for reflection usually baked into research participation is absent. Without governance processes that acknowledge and accommodate the hybrid space where research and clinical care co-exist, hybrid activities are being squeezed into poorly suited molds that risk stilting rather than facilitating research progress and good genomic medicine. Furthermore, Bertier *et al.* noted ‘*a misalignment between scientific experts’ views and legal norms of what constitutes research or care*’ and the risk that this raises of ‘*unnecessary legal and administrative repercussions on experts working in a “grey zone” at the edge of clinical care and research*’ [20].

## 3. Conclusion

To summarize, clinical care and research often represent distinct activities, guided by different principles and governed by different processes. In the context of genomics, their spheres overlap, to the point that some activities cannot meaningfully be categorized as purely clinical care, or purely research. Various aspects of genomic test interpretation intrinsically involve both the exploratory element that characteristics research, and the anticipated benefit to a particular patient that clinical care aims to achieve. Both in diagnosing a rare disease, and in discovering new rare diseases, research and clinical care have a symbiotic relationship. How can we realize the benefits of both in a world that regards them as separate?

## 4. Expert opinion

In the context of genomic testing, clinical care and research will sometimes be inseparable. Appreciating and engaging with the hybrid space where both co-exist is likely to lead to significant benefits, facilitating earlier diagnosis of rare genetic conditions, and a faster learning and discovery process as to what newly identified genetic conditions mean for patients. Explicit acknowledgment of the hybrid nature of genomic testing might also help patients have appropriate expectations from genomic testing – framing testing as involving research may mean patients are more prepared that results may be uncertain or subject to change [20].

The 2016 Chief Medical Officer’s report ‘Generation Genome’ argued the need for a rethinking of the ‘social contract’ for medical practice and research in the UK, in order to realize the potential benefits of genomics [21]. We think this should involve renegotiating existing understanding of research and clinical care as entirely separate – it does not make sense to uncouple technical identification of variants using clinical technology, from the research that might be needed to explore their clinical meaning. Similarly, when recording consent discussions relating to a genomic test, it does not make sense to ask a patient to complete separate consent exercises, one to cover the ‘clinical’ aspect, and one the ‘research’ aspect of the test. If so, we risk reducing consent to a paperwork festival, and blocking opportunities to engage fruitfully with the hybrid space where clinical practice and research co-exist.

The genomics era needs an approach to consent that recognizes the potential duality of genomic testing in encompassing both research and clinical care. Consent conversations need to adapt to reflect the interdependence of research and clinical care, perhaps representing research and clinical care as a spectrum, with opportunities for people to indicate where they sit along that spectrum in terms of their openness to taking part in the research. We appreciate that this is not a simple fix – if we do not make a clear distinction between clinical and research activities during the consent process, how can we understand the boundaries of a person’s consent, and ensure that we respect these?

One possible approach would be to focus on the areas where research and clinical care do *not* co-exist – at least this might allow some demarcation as to how far clinical consent might reasonably take us. What would constitute ‘pure research’? This might include activities that do not have the theoretical potential to directly benefit the patient having a genomic test, for example, use of their genomic data and clinical information to develop an understanding of the genetic basis of common traits like blood pressure. However, given the familial nature of genomic information, subscribing to an idea that it is the potential for direct benefit to a particular patient that defines an activity as clinical might still be complex – what if an activity might directly help a patient’s relative, but would not help the patient themselves [22]? This might be more appropriately viewed as clinical care directed outside traditional boundaries, than research – benefit would still pertain to a specific person. Another consideration is to what extent reciprocity is a reasonable expectation within a ‘contract’ of genomic testing. If a person’s variants might only be interpretable because others have made theirs available on public databases for comparison, is it reasonable to require that in turn, that that person allows anonymized recording of their variants on those same databases?

Negotiating the hybrid space between clinical practice and research, and defining its boundaries, clearly needs wider discussion. It raises controversial questions, and requires changes to existing approaches regarding consent and data management. However, we argue that the time invested in doing this would be well spent. As we prepare for widespread uptake of genomic tests, we should not accept outdated systems that cannot deal with the overlap between clinical care and research. An updated social contract that recognizes them as interdependent, and that can account for that in governance processes, is necessary in order to allow patients, the NHS, and society to realize the great potential benefits of their integration.

As previously discussed, learning health-care systems might work in this space [17], encouraging realization of the great potential for knowledge generation that genomic datasets represent, but keeping it focussed toward a goal of improving medical practice in the future. Various support groups for rare genetic conditions work in part via this principle, gathering information about the experiences of people with a particular genetic condition in order to learn about the condition and inform future clinical care. For example, the PURA Syndrome Foundation brings together families, clinicians and researchers with the aim of improving care for people with PURA syndrome. The power of their approach is demonstrated by over 300 people now having been diagnosed with PURA syndrome, and multiple research papers having been published adding to our understanding of the condition and informing clinical care [23], despite the condition only having been described in 2014 [24].

Learning health-care systems allow synergy between research and clinical care, but who should decide what they learn, and whether the benefits of doing so outweigh the burdens that these places on participants? Kass and Faden suggest actions morally required in learning health-care systems in order to respect the rights and dignity of patients: engagement about ongoing learning activities; transparency about these activities; and accountability in translating learning into practice. They argue that ‘*research ethics has operationalised the duty to respect patients almost entirely through the requirement of informed consent, and too often ethics has equated respect for patients with respect for their autonomy*’ [25]. We agree that respect for patients needs to operate more broadly. Focussing on the technicalities of detailed consent processes as the only vehicle by which respect can be shown neglects other important responsibilities to respect patients, not least, by ensuring that the learning made possible by their contribution and engagement is actually implemented in clinical practice.

## 5. Five-year view

We anticipate that the next 5 years will provide additional proof of the great benefits of clinical practice and research working in tandem. In the context of genomic testing, we need to find ways to allow these enterprises to work synergistically as a matter of course. The Chief Medical Officer’s 2016 report ‘Generation Genome’ outlined the case for rethinking the social contract for medical practice and research, and we reiterate the need for this – it does not make sense to negotiate the hybrid space where research and clinical care overlap by pretending it does not exist. Over the next 5 years, we hope new governance processes will develop that recognize the interdependence of both in providing optimal care in the genomic era. The ‘Liminal Spaces’ project is exploring health research regulation as it is experienced by stakeholders in the UK, and recently published results supporting the idea that ‘*health research regulation should continue to move away from strict, prescriptive rules-based approaches, and towards flexible principle-based regimes that allow researchers, regulators and publics to co-produce regulatory systems serving core principles*’ [26]. What principle-based regulation might look like in the context of genomic medicine remains to be worked out, but we think such an approach would be better suited to the nature of genomic testing than a formulaic rules-based approach with little scope to allow or facilitate innovation. As a starting point, we suggest that genomic testing might usefully be conceptualized as lying on a spectrum between clinical care and research, and agreeing what constitutes ‘pure research’ might give some clarity as to how far clinical consent for genomic testing can reasonably take us.

## Article Highlights

- Clinical practice and research are often conceptualized as separate, and each has different governance processes.
- In genomic testing, clinical practice and research are brought together in using exploratory, evolving scientific techniques to provide answers to clinical questions.
- There are great benefits to the hybrid space between clinical practice and research – their fusion allows discovery and diagnosis of new genetic conditions, and faster understanding of the significance of genetic variants.
- In order to allow overlapping clinical practice and research to flourish, we need to accept that they are sometimes enmeshed – governance processes that treat them as entirely distinct need updating for the genomic era.
